# Acquisition of the T790M resistance mutation during afatinib treatment in EGFR tyrosine kinase inhibitor–naïve patients with non–small cell lung cancer harboring *EGFR* mutations

**DOI:** 10.18632/oncotarget.19243

**Published:** 2017-07-12

**Authors:** Kentaro Tanaka, Kaname Nosaki, Kohei Otsubo, Koichi Azuma, Shinya Sakata, Hiroshi Ouchi, Ryotaro Morinaga, Hiroshi Wataya, Akiko Fujii, Noriaki Nakagaki, Nobuko Tsuruta, Masafumi Takeshita, Eiji Iwama, Taishi Harada, Yoichi Nakanishi, Isamu Okamoto

**Affiliations:** ^1^ Research Institute for Diseases of the Chest, Graduate School of Medical Sciences, Kyushu University, Fukuoka, Japan; ^2^ Department of Comprehensive Clinical Oncology, Faculty of Medical Sciences, Kyushu University, Fukuoka, Japan; ^3^ Department of Thoracic Oncology, National Hospital Organization Kyushu Cancer Center, Fukuoka, Japan; ^4^ Division of Respirology, Neurology, and Rheumatology, Department of Internal Medicine, Kurume University School of Medicine, Kurume, Japan; ^5^ Department of Respiratory Medicine, Kumamoto University Hospital, Kumamoto, Japan; ^6^ Department of Respiratory Medicine, Japan Community Healthcare Organization Kyushu Hospital, KitaKyushu, Japan; ^7^ Department of Thoracic Medical Oncology, Oita Prefectural Hospital, Oita, Japan; ^8^ Department of Respiratory Medicine, Saiseikai Fukuoka General Hospital, Fukuoka, Japan; ^9^ Department of Respiratory Medicine, Koga Hospital 21, Kurume, Japan; ^10^ Department of Respiratory Medicine, Steel Memorial Yawata Hospital, KitaKyushu, Japan; ^11^ Department of Respiratory Medicine, Hamanomachi Hospital, Fukuoka, Japan; ^12^ Department of Respiratory Medicine, KitaKyushu Municipal Medical Center, KitaKyushu, Japan

**Keywords:** non–small cell lung cancer (NSCLC), afatinib, acquired resistance, T790M, rebiopsy

## Abstract

The T790M secondary mutation of the epidermal growth factor receptor (EGFR) gene accounts for 50% to 60% of cases of resistance to the first-generation EGFR tyrosine kinase inhibitors (TKIs) gefitinib and erlotinib. The prevalence of T790M in *EGFR* mutation–positive patients who acquire resistance to the irreversible, second-generation EGFR-TKI afatinib has remained unclear, however. We here determined the frequency of T790M acquisition at diagnosis of progressive disease in patients with *EGFR*-mutated non–small cell lung cancer (NSCLC) treated with afatinib as first-line EGFR-TKI. Among 56 enrolled patients, 37 individuals underwent molecular analysis at rebiopsy. Of these 37 patients, 16 individuals (43.2%) had acquired T790M, including 11/21 patients (52.4%) with an exon 19 deletion of *EGFR* and 5/13 patients (38.5%) with L858R. None of three patients with an uncommon *EGFR* mutation harbored T790M. T790M was detected in 14/29 patients (48.3%) with a partial response to afatinib, 1/4 patients (25%) with stable disease, and 1/4 patients (25%) with progressive disease as the best response. Median progression-free survival after initiation of afatinib treatment was significantly (*P* = 0.043) longer in patients who acquired T790M (11.9 months; 95% confidence interval, 8.7–15.1) than in those who did not (4.5 months; 95% confidence interval, 2.0–7.0). Together, our results show that *EGFR*-mutated NSCLC patients treated with afatinib as first-line EGFR-TKI acquire T790M at the time of progression at a frequency similar to that for patients treated with gefitinib or erlotinib. They further underline the importance of rebiopsy for detection of T790M in afatinib-treated patients.

## INTRODUCTION

Several phase III trials have established epidermal growth factor receptor (EGFR) tyrosine kinase inhibitors (TKIs) as a standard first-line treatment for patients with non–small cell lung cancer (NSCLC) harboring somatic driver mutations in the *EGFR* gene [1–3]. However, all such treated patients eventually acquire resistance to these drugs, with emergence of the T790M gatekeeper mutation in the tyrosine kinase domain of EGFR accounting for 50% to 60% of instances of resistance to the first-generation EGFR-TKIs gefitinib and erlotinib [4, 5].

Afatinib is an irreversible, second-generation EGFR-TKI that is more potent than the first-generation drugs and also targets other ErbB family members such as HER2 [6]. Afatinib suppressed the growth of NSCLC cell lines harboring T790M in preclinical models [7], and it has been thought that afatinib might delay the emergence of T790M in comparison with first-generation EGFR-TKIs. We have now performed a multi-institutional study to investigate the prevalence of T790M in patients with *EGFR* mutation–positive NSCLC at the time of disease progression during treatment with afatinib as first-line EGFR-TKI therapy.

## RESULTS

**Figure 1:**
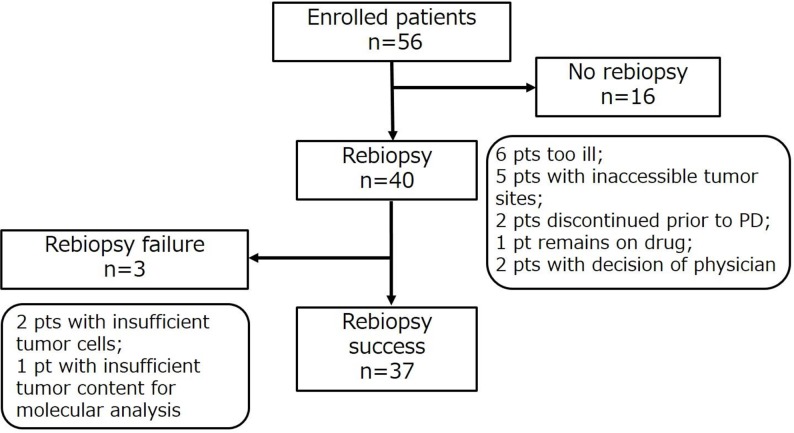
Flow chart for the study patients (pts)

**Figure 2:**
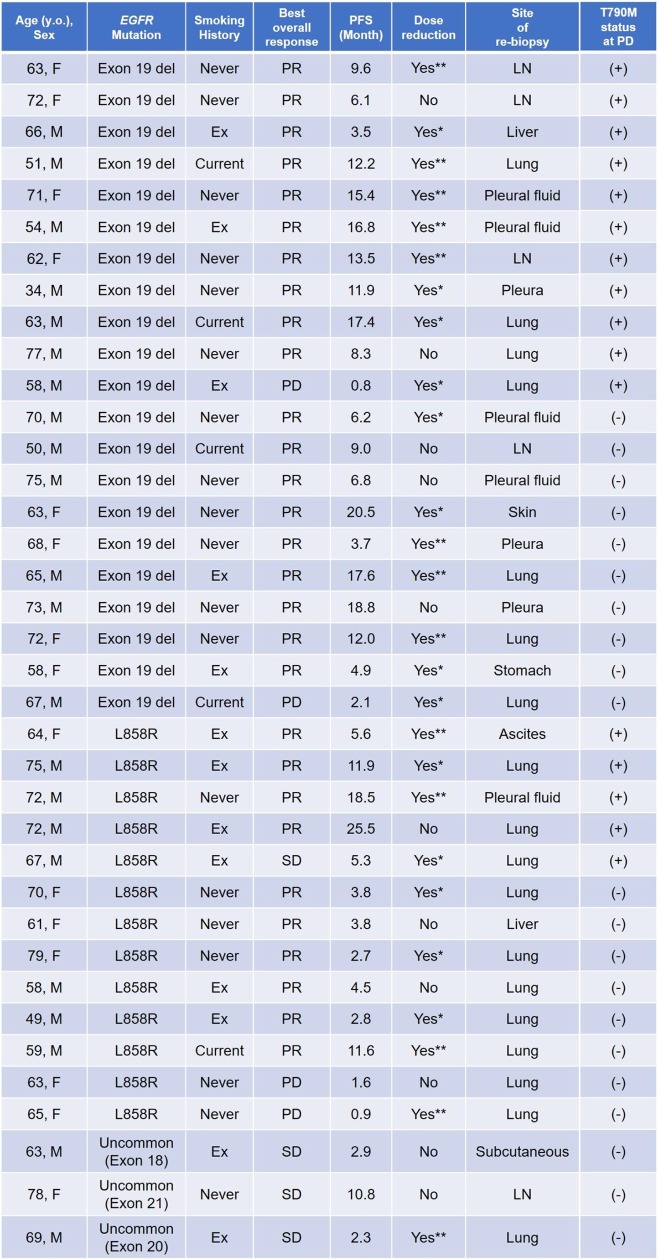
Characteristics, response to afatinib, and T790M status for the 37 patients with sufficient rebiopsy material for molecular analysis For dose reduction, Yes* indicates reduction to 30 mg/day and Yes** to 20 mg/day. Abbreviations: del, deletion; LN, lymph node.

**Figure 3:**
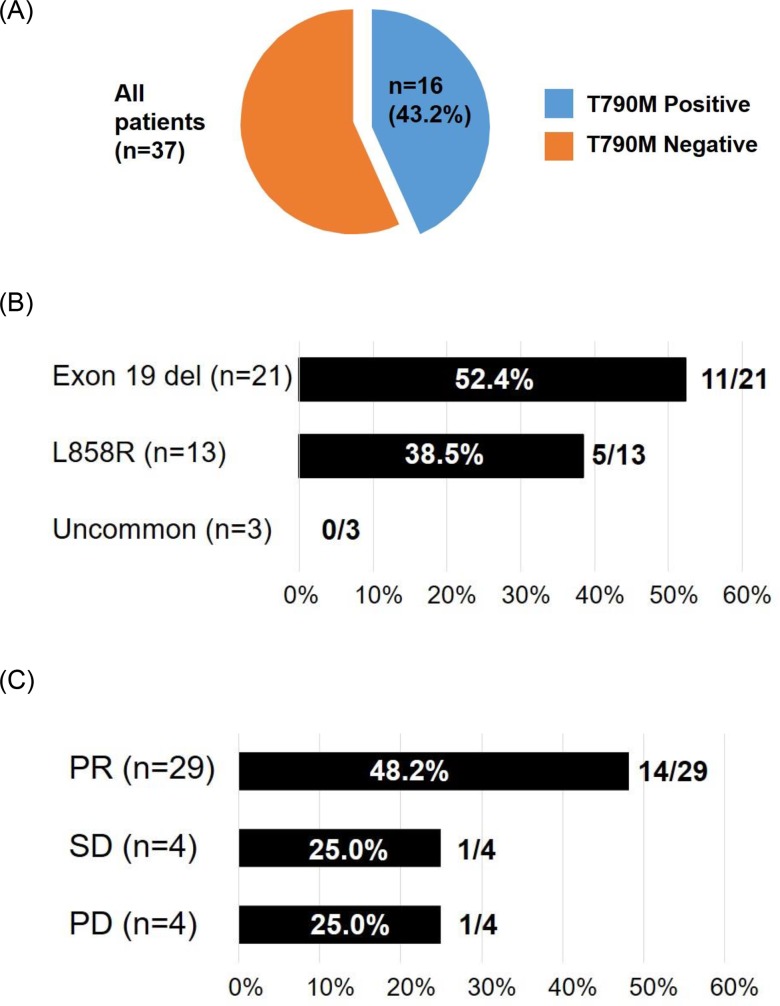
Prevalence of T790M for all patients **(A)** or according to type of activating *EGFR* mutation **(B)** or response to afatinib **(C)**.

**Figure 4:**
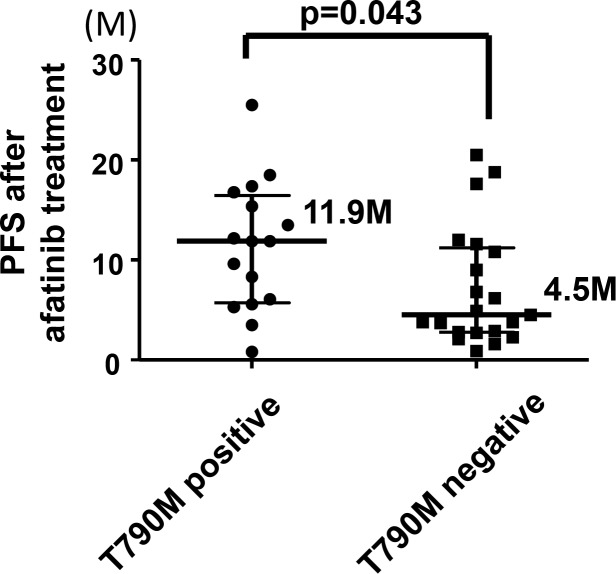
Comparison of PFS after the onset of afatinib treatment between patients who acquired T790M and those who did not Each circle or square indicates one patient. Thick horizontal bars indicate median values; error bars indicate median ± quartile deviation. The *P* value was calculated with Student’s *t* test.

Fifty-six patients who were treated with afatinib as first-line EGFR-TKI were enrolled in the study. Sixteen of these patients did not undergo rebiopsy because of concurrent illness that made the procedure infeasible (*n* = 6), inaccessible tumor sites (*n* = 5), treatment discontinuation due to adverse events (*n* = 2), the decision of the physician (based on the presence of an uncommon *EGFR* mutation) (*n* = 2), or continuation of afatinib treatment beyond progressive disease (PD) (*n* = 1). The remaining 40 patients underwent rebiopsy at the time of progression while receiving afatinib, with sufficient tissue being obtained for molecular analysis in the case of 37 patients (Figure 1). The characteristics and clinical courses of these 37 patients are shown in Figure 2. The median age of these patients was 65 years (range, 34–79), and they included 15 women. Nineteen patients had never smoked. The observed best response to afatinib was a partial response (PR) in 29 patients (29/37, 78.4%), including 19 individuals with an exon 19 deletion of *EGFR* (19/21, 90.5%) and 10 with the L858R point mutation (10/13, 76.9%). None of the three patients with uncommon *EGFR* mutations showed a response to afatinib. The dose of afatinib was reduced because of adverse events in 26 patients (26/37, 70.3%). At the time of progression, all 37 patients showed persistence of the original *EGFR* mutation, and the T790M mutation was newly detected in 16 patients (16/37, 43.2%) (Figure 3A). Eleven patients with an exon 19 deletion (11/21, 52.4%) and five patients with L858R (5/13, 38.5%) acquired T790M, whereas T790M was not detected in any of the three patients with an uncommon *EGFR* mutation (Figure 3B). Patient characteristics such as smoking history, sex, age, and performance status (PS) were not significantly associated with the emergence of T790M (Supplementary Figure 1). Dose reduction was also not associated with T790M frequency or progression-free survival (PFS) after the onset of afatinib treatment (Supplementary Figure 2). T790M was detected in 14 patients who showed a PR to afatinib treatment (14/29, 48.2%) as well as in one patient with stable disease (SD) (1/4, 25%) and one patient with PD (1/4, 25%) as the best response (Figure 3C). Median PFS after the onset of afatinib treatment was significantly (*P* = 0.043) longer in patients with T790M (11.9 months; 95% confidence interval, 8.7–15.1) than in those without it (4.5 months; 95% confidence interval, 2.0–7.0) (Figure 4).

## DISCUSSION

Emergence of the T790M mutation is the major mechanism of acquired resistance to first-generation EGFR-TKIs. Several promising new EGFR-TKIs, so-called third-generation EGFR-TKIs, including osimertinib, rociletinib, and ASP8273, have been developed. The AURA3 phase III trial revealed that the median PFS for osimertinib was significantly longer than that for platinum chemotherapy plus pemetrexed (10.1 vs. 4.4 months; hazard ratio of 0.30 with a 95% confidence interval of 0.23–0.41; *P* < 0.001) in patients with *EGFR* mutation–positive NSCLC who acquired T790M and whose disease had progressed during previous EGFR-TKI therapy, indicating that osimertinib should be a new standard treatment for this population [8]. Rebiopsy to detect T790M at the time of progression is therefore essential to determine the best subsequent treatment option for *EGFR* mutation–positive patients treated with EGFR-TKIs.

Afatinib is a second-generation, irreversible EGFR-TKI [6]. The LUX-Lung 7 phase IIb trial showed that PFS was significantly longer in patients treated with afatinib than in those treated with gefitinib as first-line EGFR-TKI [9]. This result supports the notion that afatinib is a first-line treatment option for *EGFR* mutation–positive patients. It is therefore of clinical importance to determine the prevalence of T790M in patients who experience PD during afatinib treatment. Little information has been available in this regard, however. As far as we are aware, only two studies have examined the frequency of T790M in *EGFR* mutation–positive patients treated only with afatinib [10, 11]. Wu *et al*. studied 42 patients who had tissue specimens collected after acquisition of resistance to EGFR-TKIs, among whom only 14 individuals had no treatment history with gefitinib or erlotinib. These 14 patients had received first-line afatinib until PD, and seven of them (7/14, 50%) had acquired T790M [10]. In a prospective study, Campo and others enrolled 24 patients who received treatment with afatinib as first-line EGFR-TKI. Biopsy specimens from 11 patients were subjected to molecular analysis at the time of progression, four of which (4/11, 36.4%) were positive for T790M [11]. In our study, among the 37 patients with *EGFR* mutation–positive NSCLC who were EGFR-TKI naïve, who received afatinib until diagnosis of PD, and for whom sufficient rebiopsy tissue was available for molecular analysis, the prevalence of T790M was 43.2% (16/37). Taken together, these data suggest that emergence of T790M is the major mechanism of acquired resistance to afatinib as well as that for the development of resistance to gefitinib and erlotinib [4, 5].

PFS in patients who acquired T790M in the present study was variable (Figure 4), which is consistent with the recent finding that T790M-positive cells resistant to EGFR-TKIs were heterogeneous and arose either by selection of preexisting T790M-positive clones (early emerging) or by de novo genetic evolution (late emerging) [12]. We also found that median PFS after the onset of afatinib treatment was significantly longer for patients with T790M than for those without it, consistent with the results of a previous study [13]. Preclinical models have shown that T790M-positive resistant cells grow more slowly than T790M-negative sensitive cells and suggested that longer exposure to EGFR-TKIs might promote the selective survival of T790M-positive cells [14]. This finding might thus account for the significant association between longer PFS and the emergence of T790M.

Afatinib has been found to be effective in patients with uncommon *EGFR* mutations such as exon 18/21 mutations (G719X and L861Q) and is currently a treatment option for such patients [15, 16]. However, at present, no information is available regarding the percentage of these patients who acquire T790M after a response to afatinib. We were able to analyze the clinical course of five afatinib-treated patients with uncommon mutations, two of whom did not undergo rebiopsy based on the decision of the treating physician. None of the remaining three patients showed a response to afatinib or had acquired T790M at the time of PD. Patients with uncommon *EGFR* mutations by definition are relatively rare, which will hinder determination of the prevalence of T790M acquisition during afatinib treatment in this population.

With regard to limitations of our study, its retrospective nature and case report form–based survey did not allow well-standardized measurement of PFS and response rate. In addition, the sample size was not statistically calculated, and the methods adopted to detect T790M as well as the samples used for this analysis differed among institutions.

In conclusion, we found that NSCLC patients with *EGFR* mutations who were diagnosed with PD during treatment with afatinib as a first-line EGFR-TKI acquired T790M at a frequency similar to that previously revealed for such patients treated with first-generation EGFR-TKIs. A post hoc analysis of LUX-Lung 7 suggested that overall survival was satisfactory in patients treated with afatinib followed by a third-generation EGFR-TKI [17]. Our results reinforce the importance of rebiopsy for detection of T790M in afatinib-treated patients in order to provide the opportunity for treatment with a third-generation EGFR-TKI such as osimertinib.

## MATERIALS AND METHODS

We sent case report forms to 13 institutions requesting demographic and clinical data from medical records for all patients with advanced or recurrent *EGFR* mutation–positive NSCLC who experienced PD during treatment with afatinib as first-line EGFR-TKI therapy between January 2014 and October 2016, regardless of any previous treatment with cytotoxic agents. Patients who received any other EGFR-TKI before PD or had a primary T790M mutation of *EGFR* before initial afatinib treatment were excluded. All patients were treated with afatinib at 40 mg daily, and dose interruption or dose reduction to 30 mg and 20 mg according to the decision of the treating physician was allowed. This study was approved by the institutional review board of each participating hospital and was conducted in accordance with the Declaration of Helsinki and the Ethical Guidelines for Medical Research Involving Human Subjects in Japan (dated 22 December 2014).

We obtained the following information from the case report forms: age, sex, histology, *EGFR* mutation status, disease stage, Eastern Cooperative Oncology Group PS, smoking status, the date of afatinib treatment initiation, the date of PD based on the Response Evaluation Criteria in Solid Tumors version 1.1 (RECIST), the site of rebiopsy, and T790M status at the time of PD. The primary end point of the study was the proportion of patients positive for T790M at the time of progression based on analysis of rebiopsy specimens. Secondary end points included the proportion of patients with T790M for each type of primary *EGFR* mutation (exon 19 deletion, L858R, or uncommon) and PFS after the onset of afatinib treatment.

Initial biopsy and rebiopsy specimens were analyzed for primary *EGFR* mutation and T790M status by one of the following methods: the PNA-LNA clamp (LSI Medience, Tokyo, Japan), Cycleave PCR (Takara bio, Kusatsu, Japan), Cobas *EGFR* mutation test (Roche Molecular Systems, Pleasanton, CA), Scorpion ARMS (Qiagen, Hilden, Germany), or digital PCR (Bio-Rad, Hercules, CA). Statistical analysis was performed by two-sided tests with the use of Graph Pad Prism version 5 for Windows (Graphpad Software, La Jolla, CA), and a *P* value of <0.05 was considered statistically significant.

## SUPPLEMENTARY MATERIALS FIGURES


