# Supplementary material for: Acquisition of the T790M resistance mutation during afatinib treatment in EGFR tyrosine kinase inhibitor–naïve patients with non–small cell lung cancer harboring EGFR mutations
Source: Oncotarget. 2017 Jul 12;8(40):68123–30. doi: 10.18632/oncotarget.19243 (PMC5620242; doi:10.18632/oncotarget.19243)
Supplement: Supplementary file 1 [file oncotarget-08-68123-s001.pdf]

Acquisition of the T790M resistance mutation during afatinib treatment in EGFR tyrosine kinase inhibitor–naïve patients with non–small cell lung cancer harboring *EGFR* mutations

SUPPLEMENTARY MATERIALS

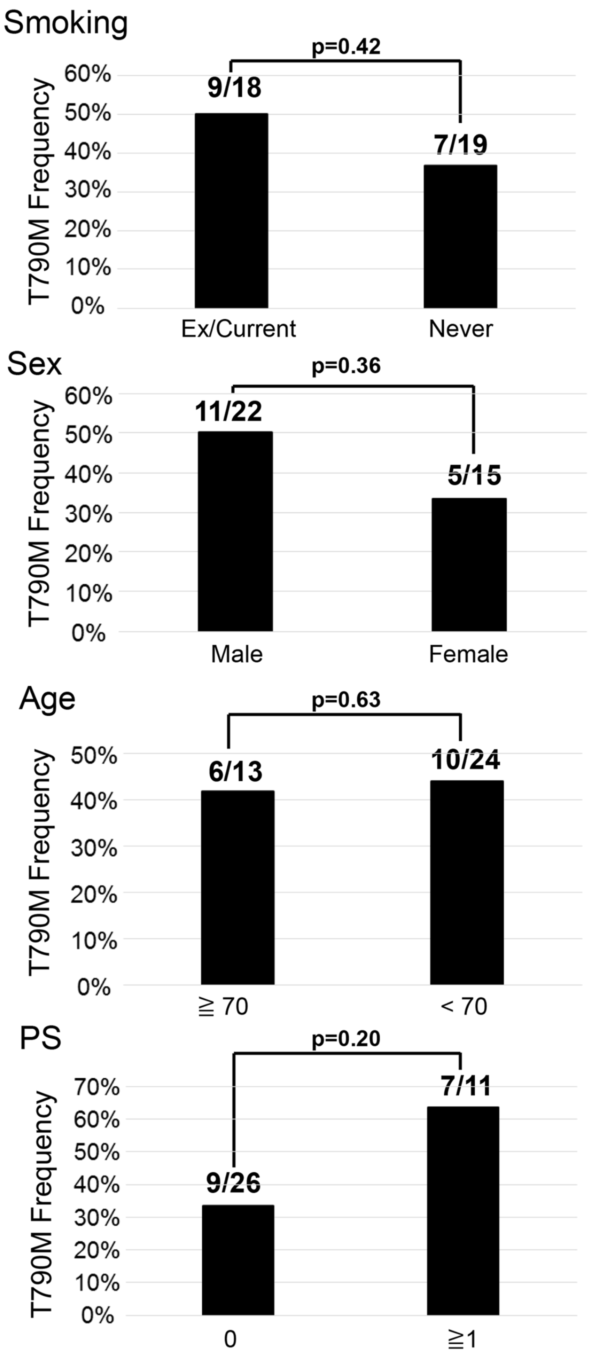

Supplementary Figure 1: Relation of patient characteristics (smoking status, sex, age, and PS) to the emergence of T790M. *P* values were calculated with the chi-square test.

(A)

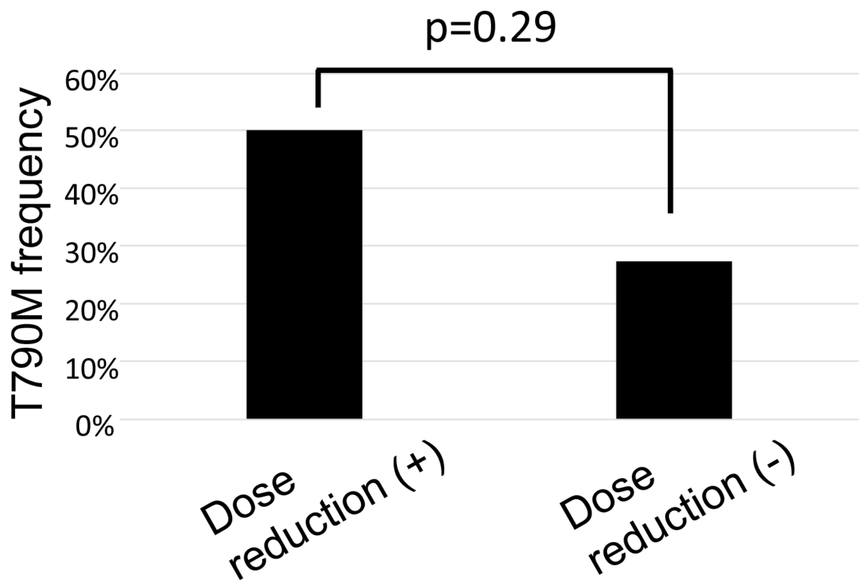

(B)

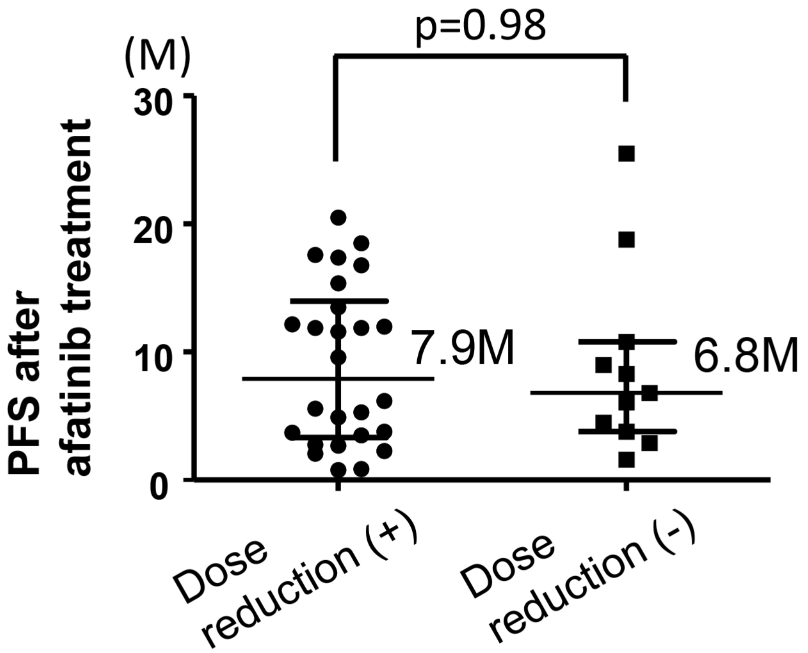

Supplementary Figure 2: Relation of dose reduction for afatinib to the emergence of T790M **(A)** or to PFS after the onset of afatinib treatment **(B).** Error bars in (B) indicate median ± quartile deviation. The P values were calculated with the chi-square test (A) or Student's t test (B).
